# Post-translational Modifications of the Protein Termini

**DOI:** 10.3389/fcell.2021.719590

**Published:** 2021-07-29

**Authors:** Li Chen, Anna Kashina

**Affiliations:** Department of Biomedical Sciences, School of Veterinary Medicine, University of Pennsylvania, Philadelphia, PA, United States

**Keywords:** N-terminome, C-terminome, acetylation, lipidation, Met-AP, arginylation, ubiquitination

## Abstract

Post-translational modifications (PTM) involve enzyme-mediated covalent addition of functional groups to proteins during or after synthesis. These modifications greatly increase biological complexity and are responsible for orders of magnitude change between the variety of proteins encoded in the genome and the variety of their biological functions. Many of these modifications occur at the protein termini, which contain reactive amino- and carboxy-groups of the polypeptide chain and often are pre-primed through the actions of cellular machinery to expose highly reactive residues. Such modifications have been known for decades, but only a few of them have been functionally characterized. The vast majority of eukaryotic proteins are N- and C-terminally modified by acetylation, arginylation, tyrosination, lipidation, and many others. Post-translational modifications of the protein termini have been linked to different normal and disease-related processes and constitute a rapidly emerging area of biological regulation. Here we highlight recent progress in our understanding of post-translational modifications of the protein termini and outline the role that these modifications play *in vivo*.

## Introduction

Post-translational modifications (PTMs) involve enzyme-mediated covalent addition of functional groups to proteins during or after synthesis. These modifications greatly increase biological complexity and are responsible for orders of magnitude of change between the variety of proteins encoded in the genome and the variety of their biological functions. Thus, PTMs dramatically expand the encoded flexibility of a living system (Marino et al., 2015). As an example, the human genome encodes 20,379 proteins, which serve as targets for 191,837 PTMs^1^. Many of these modifications occur at the protein termini, which contain reactive amino- and carboxy-groups of the polypeptide chain and often are pre-primed through the actions of cellular machinery to expose highly reactive residues. Such modifications have been known for decades, but only a few of them have been functionally characterized.

**FIGURE 1:**
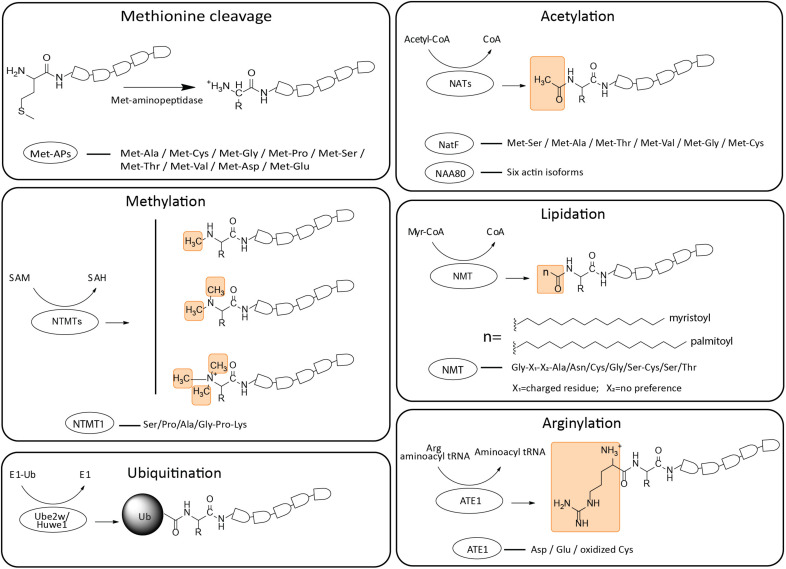
Schematic representation of structural formulas and enzymatic preferences of N-terminal modifications. The PTMs of N-termini include Met cleavage, acetylation, methylation, lipidation, arginylation and ubiquitination (yellow boxes indicate the function groups added to the termini). Corresponding recognition sites of different enzymes are listed. The responsible enzymes and their respective donor molecules are listed. Met-Aps, Met-aminopeptidases; NATs, N-terminal acetyltransferases; NTMTs, N-terminal methyltransferases; NMT, N-terminal myristoyltransferases; ATE1, arginyl transfer enzyme 1.

**FIGURE 2:**
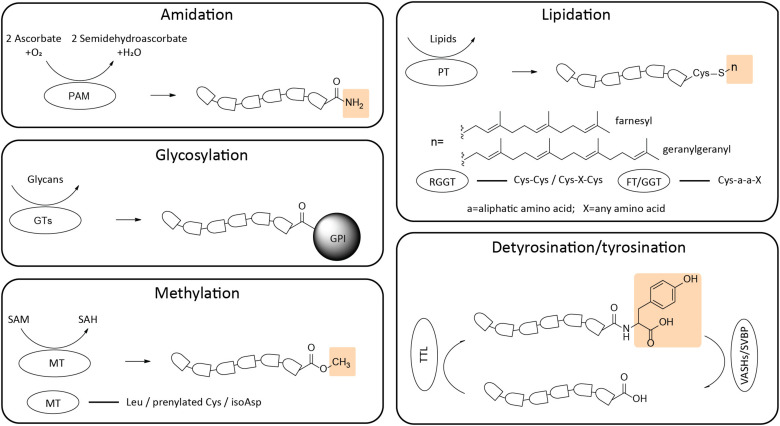
Schematic representation of structural formulas and enzymatic preferences of C-terminal modifications. The C-terminal modifications include amidation, glycosylation, methylation, lipidation and detyrosination/tyrosination (yellow boxes indicate the function groups added to the termini). Corresponding recognition sites of different enzymes are listed. PAM, peptidylglycine α-amidating monooxygenase; GTs, glycosyltransferases; MT, methyltransferase; PT, prenyltransferase; RGGT, Rab geranylgeranyl transferase; FT, Farnesyl transferase; GGT, geranylgeranyl transferase; TTL, tubulin tyrosine ligase; VASHs/SVBP, vasohibins/small vasohibin binding protein complex.

Proteins with distinct N- and C-termini possess specific biochemical properties and functions, and are collectively referred to as the protein terminome (Figures 1, 2). Since every translated protein originally contains an N-terminal Met, specialized machinery in the cell removes this residue co- or post-translationally (Figure 1). This is the critical step that makes the N-terminus accessible to many modifications that target the primary amino group of residues other than Met. Similar pre-processing can occur at the C-termini—e.g., in the case of tubulin tyrosination-detyrosination cycle, where the C-terminal Tyr originally encoded in the tubulin gene can be removed and re-ligated through the action of specialized enzymes (Murofushi, 1980; Schroder et al., 1985; Aillaud et al., 2017; Nieuwenhuis et al., 2017; reviewed in Nieuwenhuis and Brummelkamp, 2019; Figure 2). Some PTMs at the protein termini can be more abundant than PTMs targeting the internal sites; for example, N-terminal acetylation can modify 80–90% of soluble human proteins and 50–70% of yeast proteins (Arnesen et al., 2009; Van Damme et al., 2011b). Terminal modifications target over 90% of the mammalian proteome and are essential for a variety of biological functions and processes such as protein sorting, membrane integration, cellular signaling, protein transport, enzyme activity, and formation of protein complexes. Thus, terminal modifications represent an important contribution to proteomic diversity and complexity.

In this review we discuss the major types of N- and C-terminal modifications that have been characterized to date, focusing on eukaryotic and mammalian systems.

## N-Terminal Post-Translational Modifications

### N-Terminal Removal of Amino Acid Residues and Signal Peptides

Since the ribosome recognizes the AUG codon as the translation initiation site, all naturally synthesized proteins contain an initiator Met at their N-terminus. This Met in itself is a reactive residue that can be modified by acetylation (Van Damme et al., 2012), as well as potentially serve as a target for other PTMs, e.g., as oxidation target for reactive oxygen species, though biological examples of such modifications have yet to be described. In many eukaryotic proteins the initiator Met is co- or post-translationally removed by Met-aminopeptidases (Met-APs), to expose the second residue in the sequence, which can in turn be removed or targeted by other PTMs.

Eukaryotic cells contain two of major Met-APs, Met-AP1 and Met-AP2 (Arfin et al., 1995; Li and Chang, 1995). These enzymes remove the Met preceding small and uncharged amino acid residues (e.g., Gly, Ala, Ser, Cys, Pro, Thr, and Val) (Boissel et al., 1985; Tsunasawa et al., 1985; Flinta et al., 1986; Ben-Bassat et al., 1987; Xiao et al., 2010; Wingfield, 2017). While the exact biological functions of these enzymes have not been characterized in detail, the overall activity of these enzymes is required for cell viability: their deletion leads to lethality in yeast (Li and Chang, 1995), and inhibition of their activity causes cell death in mammalian cell cultures (Towbin et al., 2003).

While no other Met-APs have been described, the repertoire of proteins found *in vivo* with removed N-terminal Met increasingly suggests that other classes of Met-APs must exist in cells, with specificity for residues in the second position that goes beyond the limited list of those recognized by Met-AP1 and Met-AP2. For example, cytoplasmic actins are processed by removal of N-terminal Met preceding negatively charged residues (Asp or Glu), which occurs after Met acetylation (Redman and Rubenstein, 1981; Rubenstein and Martin, 1983; Martin and Rubenstein, 1987; Van Damme et al., 2012). The enzyme mediating this removal has been biochemically enriched but never definitively identified (Sheff and Rubenstein, 1992). Emerging mass spectrometry data suggest that initiator Met removal may occur even outside this residue specificity, suggesting that other Met-APs with different target sites might also exist in cells. Identification of these enzymes constitutes an exciting potential direction of research.

Removal of N-terminal Met exposes the next residue in the sequence to other, non-Met aminopeptidases, which can catalyze sequential removal of amino acid residues from the N-terminus unless it becomes structurally or chemically protected. According to MEROPS database (Rawlings, 2009), there are currently 269 aminopeptidases of different specificity found across the kingdoms of life^2^. While some of these aminopeptidases have been characterized in different systems, the global contribution of this processing to the functional N-terminome has not been systematically addressed.

Another type of N-terminal protein processing involves removal of signal peptides, e.g., from proteins secreted through the ER or targeted to other intracellular compartments. This cleavage exposes reactive residues on the N-termini that are further targeted by various modifications, both in the intracellular compartments and in the extracellular space.

Both signal peptide cleavage and the action of endopeptidases generate neo N-termini that can be subjected to a multitude of different N-terminal modifications (Gevaert et al., 2003; Auf dem Keller et al., 2013). For example, an estimated 64 to 77% of identified termini in human erythrocytes and platelets differ from the genetically encoded termini and originate largely through proteolytic cleavage (Lange et al., 2014; Prudova et al., 2014). Collectively, these events increase the complexity of the N-terminome *in vivo* and the variety of N-terminal modifications on the same protein during different physiological processes *in vivo*.

#### Acetylation

N-terminal acetylation is one of the most abundant protein modifications in eukaryotes. This modification involves the transfer of an acetyl moiety from acetyl-CoA to the α-amino group of a nascent polypeptide or the fully synthesized protein. Even though some acetylation occurs directly on the initiator Met, most of these events in eukaryotes require Met removal and target the second or third residue in the protein sequence (Polevoda and Sherman, 2000). 80–90% of all proteins in human and 50–70% in yeast are N-terminally acetylated (Arnesen et al., 2009).

N-terminal acetylation is catalyzed by one of seven N-terminal acetyltransferases (NATs) (NatA to NatF and NatH). All NATs are oligomeric complexes composed of at least one catalytic subunit and one or more auxiliary subunit(s), including a unique ribosomal anchor, which contribute to substrate specificity and interactions with nascent polypeptides (Ree et al., 2018). Five of the NATs (NatA to NatE) act co-translationally on nascent peptides emerging from the ribosome. NatF binds to the Golgi membrane and acetylates transmembrane proteins (Chen et al., 2016), most likely in a post-translational manner (Aksnes et al., 2015). Different NATs exhibit different target site specificity. Out of the three major NATs responsible for the majority of acetylations in eukaryotes, the NatA complex, acetylates N-termini starting with Ala, Cys, Gly, Ser, Thr or Val following the removal of the preceding initiator Met. Of note, removal of the auxiliary subunit from this enzyme complex changes its specificity to target the acidic N-termini (Polevoda et al., 1999; Arnesen et al., 2009; Van Damme et al., 2011a), though it is unclear whether this mechanism regulates N-terminal acetylation *in vivo*. The NatB complex acetylates the initiator Met preceding Asn, Asp, Gln, or Glu (Polevoda and Sherman, 2003). NatC targets the initiator Met that precedes Ile, Leu, Phe, or Trp. Other NATs also exhibit specific acetylation signatures, and sometimes show preference toward specific classes of proteins (e.g., histones in the case of NatD). These specificities are not absolute, and some overlapping protein targets have been observed between different NATs.

In addition to these broader specificity NATs, some additional N-terminal acetyltransferases exist that are uniquely specialized to target specific proteins *in vivo*. The recently identified NAA80 uniquely recognizes actin (Drazic et al., 2018). NAA80 acts post-translationally, recognizing fully synthesized and folded actin and targeting all six mammalian actin isoforms in unique processing steps that occur differently in muscle and non-muscle actins.

Actin N-terminal processing constitutes a striking example of the complexity of the machinery mediating N-terminal acetylation and removal of specific residues in a protein. Non-muscle actins, containing a string of negatively charged residues following the initiator Met (DDD in beta actin and EEE in gamma actin), are co-translationally acetylated on the initiator Met *via* NatB, followed by removal of the acetylated Met by an unidentified actin *N*-acetyl-aminopeptidase (ANAP) (Sheff and Rubenstein, 1992). Muscle actins, typically containing a Cys in the second position, are processed by Met-AP1/2 to remove the N-terminal Met, followed by Cys acetylation, likely by NatA, and acetylated Cys removal by ANAP, to expose the acidic residue in the third position. In all cases, this acidic residue is then recognized by NAA80 in a posttranslational manner after actin’s emergence from the ribosome and folding. Notably, NAA80 appears to have uniquely coevolved with actin to enable this preferential recognition, facilitated by the negatively charged actin’s N-terminus, as well as the actin-profilin complex (Rebowski et al., 2020). It appears likely that other proteins may also have dedicated acetyltransferases that are yet to be discovered.

While the exact biological role of N-terminal acetylation is still being investigated, studies of individual proteins, as well as knockouts of individual *N*-acetyltransferases, enable some insights into their functions. Acetylation of fetal hemoglobin promotes subunit interactions in the hemoglobin tertiary complex (Manning and Manning, 2001), while tropomyosin requires acetylation for binding to actin (Urbancikova and Hitchcock-DeGregori, 1994). NAT knockouts in yeast lead to systemic growth and mating defects (Polevoda et al., 1999). In the case of actin, NAA80 knockout results in disrupted cytoskeleton structure and dynamics, including increased ratio of filamentous to globular actin, increased filopodia and lamellipodia formation, and accelerated cell motility (Drazic et al., 2018).

To date, no N-terminal deacetylases that globally remove N-terminally added acetyl groups have been identified, and thus N-terminal acetylation of proteins is believed to be irreversible.

#### Lipidation

N-terminal lipidation refers to the transfer of fatty acids from acyl-CoA to the N-terminal amino acid residue. The most extensively studied type of lipidation is called myristoylation, which involves the addition of myristic acid to the N-terminal glycine (Gly) of the target protein. This chemical reaction is catalyzed by N-terminal myristoyltransferases (NMTs), which modify proteins in a co-translational manner in most of the cases (Glover et al., 1997; Varland et al., 2015) by targeting the amino acid residue N-terminally exposed after the removal of the initiator Met.

Two NMTs exist in mammalian systems, NMT1 and NMT2 (Gordon et al., 1991). These enzymes recognize an N-terminal 5-residue consensus sequence, which always starts with a Gly, followed by a charged residue in the second position, Ala/Asn/Cys/Gly, or Ser in the fourth position, and a preferred Cys/Ser, or Thr in the fifth position. There is no preference for the third position. Aromatic residues and Pro in the second position and/or Pro in the fifth position are incompatible with myristoylation (Towler et al., 1987; Martin et al., 2011).

N-terminal myristoylation targets an estimated 0.5% of cellular proteins (Thinon et al., 2014). Global profiling of N-terminal post-translational myristoylation found 40 substrates of NMTs in human cells. These proteins distribute in most of the organelles and are responsible for a variety of cell functions including apoptosis, DNA damage and repair, cell cycle regulation, and others. While our understanding of the biological role of myristoylation is still far from complete, studies demonstrate its key involvement in immune response (Udenwobele et al., 2017), protein turnover (Timms et al., 2019), G-protein signaling (Linder et al., 1993), and targeting of proteins to the membranes in different intracellular compartments (Resh, 2006; Baekkeskov and Kanaani, 2009). Huntingtin protein (HTT) is post-translationally myristoylated following the cleavage by caspases, and disruption of this myristoylation process on HTT fragment might be involved in the pathophysiology of Huntington disease (Martin et al., 2014).

Pro-apoptotic protein fragments with exposed N-terminal Gly undergo extensive myristoylation events in apoptotic cells (Zha et al., 2000; Utsumi et al., 2003). Strictly speaking, such myristoylation is not exactly N-terminal, since it occurs on pre-proteolyzed peptides, however, it is mediated by the same enzymes and thus belongs in this overview of NMT-mediated protein regulation. By definition, such myristoylation in apoptotic cells is always post-translational, targeting fully synthesized and proteolyzed proteins. A prominent example is BID, a pro-apoptotic Bcl-2 protein containing only the BH3 domain, which is cleaved by caspase 8, generating a truncated 15-kd fragment (tBID) that translocates to the mitochondria within 1 hour. Myristoylation of tBID fragment acts as an activating switch, enhancing BID-induced release of cytochrome c and cell death (Zha et al., 2000). N-myristoylation of p21-activated kinase 2 (PAK2) targets its C-terminal fragment (ctPAK2), facilitating its relocation from the cytosol to the plasma membrane and membrane ruffles to maintain the normal apoptosis (Vilas et al., 2006).

Another type of N-terminal lipidation is palmitoylation, the addition of a palmitoyl group that can occur both N-terminally and internally in the protein. In the latter case, palmitoylation targets the side chains of Cys residues. N-terminal palmitoylation is far less abundant, and only a few examples of protein regulation by this modification have been identified. A palmitic acid-modified form of Sonic Hedgehog plays a role in regulating its intracellular functions (Pepinsky et al., 1998). Palmitoylation of the alpha subunit of the heterotrimeric G protein on the N-terminal Gly facilitates G-protein-dependent activation of adenylyl cyclase (Kleuss and Krause, 2003). Further studies are needed to uncover additional biological roles of this modification.

#### Methylation

N-terminal methylation catalyzes the transfer of a methyl group from *S*-adenosylmethionine (SAM) to the exposed N-terminal α-amino group after the initiator Met cleavage. The properties of methylated proteins differ by the degree of residue methylation. Monomethylation slightly increases basicity of the α-amino group, and also introduces minor steric hindrance that may reduce its reactivity. However, dimethylation and trimethylation generate a permanent positive charge on the N-terminal amino group. The reversed electric charge properties cause the loss of nucleophilicity generated by α-amino nitrogen. Up until now, no N-terminal demethylase has been found and this process is believed to be irreversible.

Although this modification was found more than 40 years ago, its physiological function was not clear until the discovery of its importance in protein-DNA interactions (Chen et al., 2007). In 2010, two different groups identified N-terminal methyltransferases (NTMTs) in yeast and human, respectively (Tooley et al., 2010; Webb et al., 2010). Two NTMTs are identified to date: NTMT1 is a tri-methyltransferase responsible for mono-, di-, and trimethylation of its substrates whereas NTMT2 is primarily responsible for monomethylation (Varland et al., 2015).

NTMT1, highly conserved from yeast to humans, recognizes the substrates with consensus sequence X-Pro-Lys (X = Ser/Pro/Ala/Gly). However, a recent study found that NTMT1 has broader recognition of peptides *in vitro*, where X can also include Phe, Tyr, Cys, Met, Lys, Arg, Asn, Gln, or His, suggesting that this enzyme, under different conditions, can also have broader substrate specificity *in vivo* (Petkowski et al., 2012). A crystal structure revealed that NTMT1 contains a typical methyltransferase Rossmann fold which is composed of a seven-strand β-sheet and five α-helices. Two α-helices (α6 and α7) pack on one side of the β-sheet, and the other three α-helices (α3,α4, and α5) pack on the other side of the β-sheet. Besides its highly conserved Rossmann fold, NTMT1 has two unique structural elements distinct from other methyltransferases: a β hairpin inserted between strand β5 and helix α7 and an N-terminal extension consisting of two α-helices (α1 and α2). These two unique structures, which are involved in substrate binding, contribute to substrate specificity (Dong et al., 2015).

Many N-methylated proteins are components of large multisubunit complexes, suggesting a role of N-methylation in the regulation of protein-protein interactions (Stock et al., 1987). N-terminal α-methylation on Ser2 of RCC1, the only known guanine nucleotide-exchange factor for the Ran GTPase, is indispensable for stable chromatin association and normal mitosis (Chen et al., 2007). During mitosis, CENP-A recruits the constitutive centromere associated network (CCAN) complex to govern the centromere function. N-terminal trimethylation on Gly2, followed by removal of the initiator Met, keeps the appropriate function of CENP-A, and loss of trimethylation can lead to lagging chromosomes and spindle pole defects (Sathyan et al., 2017). Mass spectrometric analysis revealed that the N-terminal Ala of PARP3 [poly(ADP-ribose) polymerase 3] is heavily methylated; however, the function of this methylation is not yet known (Dai et al., 2015).

N-terminal methyltransferases play important roles in maintaining proper cell function. Loss of the N-terminal methyltransferase NRMT1 disrupts the DNA damage repair and promotes mammary oncogenesis (Bonsignore et al., 2015a). The NRMT1 knockout (Nrmt1−/−) mice show high mortality after birth, and the surviving minority of these mice exhibit a variety of defects including decreased body size, female-specific infertility, kyphosis, decreased mitochondrial function, and early-onset liver degeneration (Bonsignore et al., 2015b).

#### Arginylation

Protein arginylation was originally discovered in 1963, when it was found that ribosome-free extracts from cells and tissues exhibit prominent incorporation of specific radioactive amino acids (Kaji et al., 1963a,b). In eukaryotes, this catalytic reaction transfers Arg from aminoacyl tRNA to the target protein independently of the ribosome (Kashina, 2015). The enzyme mediating this reaction was first cloned and characterized in yeast, and termed arginyl transfer enzyme 1 (ATE1), or arginyltransferase (Balzi et al., 1990). To date, no stable binding partners or cofactors modulating ATE1 activity have been characterized, with the exception of Liat1 that was shown to bind to ATE1 and stimulate its ability to N-terminally arginylate a model substrate *in vitro* (Brower et al., 2014). The *Ate1* gene exists in nearly all eukaryotes [with the exception of two protozoan species (Jiang et al., 2020)], and plants have an additional gene, *Ate2*, which is believed to have arisen through gene duplication and carries a redundant biological function (Graciet and Wellmer, 2010; Domitrovic et al., 2017). The *Ate1* gene in human and mouse encodes four isoforms, generated by alternative splicing (Rai and Kashina, 2005). Additional two isoforms, containing a tandem assembly of normally alternatively spliced exons, have been reported in one study (Hu et al., 2006), but the existence of these two isoforms has not been corroborated in studies by other groups.

ATE1 preferentially targets the unacetylated acidic N-terminal residues, including Asp and Glu (Wang et al., 2018), and has also been found to target oxidized Cys (Hu et al., 2008; Sriram et al., 2011). Targeting of unmodified Cys and other N-terminal amino acid residues has also been reported (Wong et al., 2007), likely at far lesser frequency than that of the “canonical” sites, and this targeting was found to differ between different ATE1 isoforms (Rai et al., 2006; Wang et al., 2019). While early studies did not observe any apparent consensus sequence for arginylation, recent high throughput analysis of arginylation using peptide arrays and advanced prediction tools suggested the existence of a consensus motif that may potentially be used to predict arginylation sites *in vivo* (Wang et al., 2019). Furthermore, recent studies uncovered the ability of ATE1 to arginylate the acidic side chains of Asp and Glu located internally in the protein sequence, expanding the role of this enzyme beyond the N-terminome (Wang et al., 2018).

ATE1-mediated arginylation has been initially characterized as part of the N-degron (N-end rule) pathway that relates the protein’s half-life to the identity of the N-terminal amino acid residue (Bachmair et al., 1986; Varshavsky, 2019). In this pathway, the N-terminally arginylated proteins are recognized by specific E3 ligases of the ubiquitin–proteasome system (UPS), which then ubiquitinate a nearby Lys for the follow-up degradation. However, many arginylated proteins do not contain an accessible Lys for this targeting and thus remain metabolically stable after arginylation. Moreover, global analysis of protein arginylation targets suggest that regulation of protein stability constitutes only a small subset of ATE1’s *in vivo* functions (Wong et al., 2007). To date, only a few proteins regulated by ATE1-dependent degradation have been uncovered, including members of the RGS family (Lee et al., 2005), and Apetala2/Ethylene Response Factors in plants (Gibbs et al., 2011; Licausi et al., 2011; Phukan et al., 2017). At the same time, non-degradatory targets of ATE1 have also been reported, including calreticulin (Carpio et al., 2010), phosphoribosyl pyrophosphate synthase (Zhang et al., 2015), and non-muscle β-actin (Karakozova et al., 2006). β-actin is N-arginylated at the third residue from the N-terminus, Asp3, after removal of the initiator Met and Asp2, and this arginylation is believed to regulate its functions in cytoskeleton maintenance and cell motility (Saha et al., 2010; Pavlyk et al., 2018), however, these mechanisms have not yet been fully characterized. Arginylated calreticulin is less susceptible to proteasomal degradation compared to the non-arginylated one (Goitea and Hallak, 2015), and its arginylation, following retrotranslocation of calreticulin from the ER into the cytosol, has been shown to increase apoptotic response (Comba et al., 2019). Further studies are underway and will eventually unravel the full complexity of the N-terminal arginylome.

#### Ubiquitination

N-terminal ubiquitination involves the addition of a ubiquitin moiety to the free α-amino group of the first residue of a protein. The N-terminal ubiquitin may serve as a target for polyubiquitination, which is a well-known degradation signal recognized by the 26S proteasome complex. The addition of ubiquitin to the N-terminus of a target protein requires the same enzymatic machinery as ubiquitination of internal residues in the protein sequence, including ubiquitin activating, conjugating, and ligating enzymes.

N-terminal ubiquitination was first discovered by Ciechanover’s lab (Breitschopf et al., 1998), which observed that ubiquitin-dependent degradation of myogenic transcriptional switch protein MyoD was not significantly affected by substitution of all nine internal Lys residues, but was abolished by chemical modification of the α-amino group (Breitschopf et al., 1998). Direct evidence of N-terminal protein ubiquitination was obtained later by mass spectrometry, using tumor suppressor protein p16INK4a and the human papillomavirus oncoprotein-58 E7 (Ben-Saadon et al., 2004). Recently, it was found that α-synuclein and a tau tetra-repeat domain can be N-terminally ubiquitinated *in vitro*. This ubiquitination affects aggregation properties, and was proposed to enable targeting of the modified α-synuclein and a tau tetra-repeat domain by the proteasome, suggesting a role of N-terminal ubiquitination in the removal of amyloidogenic proteins (Ye et al., 2020).

Until now, two enzymes in the ubiquitin pathway, Ube2w and Huwe1, are reported to have the ability for N-terminal ubiquitination. Ube2w is an E2 ligase that conjugates ubiquitin to the N-terminal residue of Ataxin-3 and tau protein. Comparison of the active sites of Ube2w and classical E2s shows distinctive features, which make the Ube2w better suited for a neutral α-amino group rather than a positively charged Lys side chain (Scaglione et al., 2013; Tatham et al., 2013). Huwe1 is the ubiquitin E3 ligase responsible for MyoD N-terminal ubiquitination. It was reported to play an important role in the nervous system, including neural progenitor proliferation, differentiation, cell migration, axon development, and inhibitory neurotransmission (Giles and Grill, 2020). Loss of function of Ube2w increases the susceptibility to early postnatal lethality and defects in skin, immune, and Male reproductive systems (Wang et al., 2016).

#### N-Degron

Earlier studies of protein degradation revealed that protein’s N-terminus often determines the protein’s half-life, leading to the discovery of the “N-end rule pathway” (Bachmair et al., 1986) (later termed N-degron pathway) that related ubiquitin-dependent protein degradation to the identity of the N-terminal residue [see, e.g., (Varshavsky, 2011; Eldeeb and Fahlman, 2016; Winter et al., 2021) for an overview]. Since all proteins initially contain a Met at the N-termini, generation of the N-degrons requires additional processing that can include the action of the Met-APs, discussed earlier in this review, or regulated proteolysis that can expose a destabilizing N-terminal residue (Rao et al., 2001; Piatkov et al., 2012, 2014). Additional pathways can include N-terminal deamidation of Asn or Gln, followed by their Ate1-mediated arginylation (termed secondary and tertiary steps of the N-end rule pathway, see Varshavsky (2019) for a recent review). In eukaryotes, N-terminal acetylation can serve as a degradation signal (Shemorry et al., 2013; Varshavsky, 2019), even though other studies propose that the hydrophobicity of the N-terminus, rather than acetylation, might serve a defining role in this mechanism (Eldeeb et al., 2019). More recently, the scope of the N-degron pathway was expanded from solely ubiquitin-proteasome degradation to autophagy and lysosomal pathways (Cha-Molstad et al., 2015, 2017; Yoo et al., 2017, 2018; Shim et al., 2018; Ji et al., 2019; Winter et al., 2021). Through all these processes, N-degrons are believed to regulate protein quality control as an integral part of key intracellular processes (Tasaki and Kwon, 2007; Gibbs et al., 2016; Ji and Kwon, 2017; Kwon and Ciechanover, 2017; Timms and Koren, 2020).

## C-Terminal Post-translational Modifications

### Amidation

Carboxy-terminal α-amidation—the addition of an amide group to the end of the polypeptide chain—is an important post-translational modification found widely in cells. The α-amide group neutralizes the negatively charged C-termini, preventing ionization of the C-terminus and improves receptor binding ability of proteins and peptides (Cui et al., 2013; Marino et al., 2015). The most representative α-amidated peptides are found in the nervous and endocrine systems, including neurokinin A, calcitonin, and amylin (Kim and Seong, 2001).

The α-amidation reaction is catalyzed by peptidylglycine α-amidating monooxygenase (PAM), a bifunctional enzyme composed of peptidylglycine-α-hydroxylating monooxygenase (PHM: E.C. 1.14.17.3) and peptidyl-α-hydroxyglycine-α-amidating lyase (PAL: E.C. 4.3.2.5) domains. The first step of amidation is catalyzed by PHM, a dicopper and ascorbate-dependent monooxygenase, which hydroxylates peptidylglycine generated from precursor proteins by sequential endo- and exoproteolysis. The hydroxylated product of this reaction, peptidyl-α-hydroxyglycine, is N-oxidatively cleaved by PAL, a zinc-bounded lyase (Chufán et al., 2009). X-ray structure analysis of PHM catalytic core (Prigge et al., 1997) revealed that this core is organized into N- and C-terminal domains of about 150 residues connected by a linker peptide. Each domain contains nine β strands and binds one copper ion (Cu_M_ and Cu_H_). Crystal structure shows that PAL folds as a six-bladed β-propeller. The active site is composed of a Zn(II) ion, coordinated by three histidine residues; the substrate binds to the active site through its α-hydroxyl group linked to the Zn(II) ion (Chufán et al., 2009).

Over half of all biologically active peptides and peptide hormones are C-terminally α-amidated, which is essential for their full biological activities (Kim and Seong, 2001). In most cases, this structural feature is essential for receptor recognition, signal transduction, and ligand binding. Mutation of the PAM gene leads to larval lethality in *Drosophila* and embryonic lethality in mice (Kolhekar et al., 1997; Czyzyk et al., 2005).

### Glycosylation

Glycosylation involves addition of carbohydrate moieties to proteins *in vivo*. Protein glycosylation plays an important overall role in protein maturation and sorting, affecting a wide variety of normal and pathological functions [e.g., (Varki, 2017; Reily et al., 2019) for recent reviews]. Protein glycosylation is a complicated multistep process that can target different chemical groups in proteins by addition of different sugar moieties (glycans) and utilizes around 200 glycosyltransferases (GTs) with different specificities. Common types of glycosylation include N-linked glycosylation, O-linked glycosylation, addition of phosphorylated glycans and glycosaminoglycans to different amino acid residues in protein midchain sites, as well as C-terminal addition of glycosylphosphatidylinositol (GPI).

One well-described example of C-terminal glycosylation involves addition of GPI to serve as a lipid anchor for protein binding to the cell surface. GPI is conjugated to the protein C-terminus *via* an amide bond between the carboxyl group and an amino group of the terminal ethanolamine phosphate. This is a multi-step process, in which the core GPI is assembled on the endoplasmic reticulum (ER) membrane and then transferred to the precursor proteins immediately after their ER translocation. Following this, the nascent GPI-linked protein undergoes several modification steps, including addition of side glycans in the ER and the Golgi apparatus (Homans et al., 1988; Kinoshita, 2020). The final products are transported to the plasma membrane.

In humans, at least 150 GPI-anchored proteins have been identified, and these proteins play a variety of different roles in cells, serving as receptors, adhesion molecules, enzymes, transporters, and protease inhibitors (Kinoshita, 2016). Complete loss or severe reduction in GPI biosynthesis lead to early embryonic lethality in mice (Alfieri et al., 2003; Reily et al., 2019). Inherited GPI deficiency causes neurological problems, including seizures, developmental delay/intellectual disability, cerebral atrophy and hypotonia (Knaus et al., 2018; Bellai-Dussault et al., 2019).

Another type of C-terminal glycosylation involves perforin, a pore-forming cytolytic protein found in the granules of cytotoxic T lymphocytes and natural killer cells. Perforin forms transmembrane pores on the target cell membrane, which allow for the passive diffusion of granzymes into the target cell and lead to cell apoptosis (Podack et al., 1985; Stinchcombe et al., 2006). N-linked glycosylation of the perforin’s C-terminal Asn549 plays a vital role in protecting cytotoxic lymphocytes from perforin cytotoxicity by preventing perforin pores formation prior to granule exocytosis (House et al., 2017).

### Lipidation

Protein C-terminal lipidation, also called prenylation, refers to the addition of multiple isoprene units to cysteine residues close to the C-termini of proteins. For an estimation, about 2% of the total proteins in mammalian cells are prenylated (Epstein et al., 1991; Jiang et al., 2018). Two types of lipid groups, farnesyl and geranylgeranyl, target the cysteine at the C-termini of protein with conserved motifs CaaX CC and CXC (C = cysteine, a = an aliphatic amino acid, X = any amino acid). The majority of prenylated proteins undergo geranylgeranylation (Epstein et al., 1991). This modification is thought to be irreversible, as no enzymes have been found to be responsible for de-prenylation of intact proteins. However, studies revealed that a prenylcysteine lyase exists in lysosomes and is responsible for thioether bond of prenylcysteines cleavage during degradation of prenylated proteins (Tschantz et al., 1999, 2001).

There are three members of prenyltransferase (PT) family in eukaryotes, including farnesyl transferase, geranylgeranyl transferase, and Rab geranylgeranyl transferase. Farnesyl transferase (FT) catalyzes 15-carbon farnesyl group transfer to target protein; geranylgeranyl transferase (GGT) is responsible for transfer of 20-carbon geranylgeranyl group. After these two transferases target Cys within the C-terminal CaaX motif, the C-terminal aaX is further processed through endoplasmic reticulum (ER) protease cleavage, followed by carboxylmethylation on prenylated cysteine residue in the ER (Boyartchuk et al., 1997; Dai et al., 1998). The Rab geranylgeranyl transferase (RGGT) recognizes Rab protein CC or CXC motifs and transfers geranylgeranyl groups on the C-terminal double cysteine (Jiang et al., 2018). Crystal structures analyses of GGT and FT revealed that the active sites of these two enzymes are composed of conserved aromatic residues that bind hydrophobic isoprene units, located in the β subunits. Zn(II) ions are required for their enzymatic activity (Park et al., 1997; Taylor et al., 2003).

Prenylation modulates protein membrane localization and protein-protein interaction. Defects in isoprene units biosynthesis or regulation can lead to cancer, cardiovascular and metabolic diseases, and neurodegenerative disorders (Xu et al., 2015). For example, lamin B requires farnesylation to assemble into the lamina for its association with the nuclear membrane during mitosis (Sinensky, 2011). Defects in lamin B1 prenylation can lead to abnormal brain development, and non-farnesylated form of lamin B1 causes mouse death soon after birth (Mical and Monteiro, 1998; Jung et al., 2013).

### Methylation

C-terminal methylation is an important branch of protein methylation, involving the enzymatic transfer of methyl groups from *S*-adenosylmethionine (SAM) to proteins. C-terminal methylation targets several amino acid residues, including Leu, prenylated Cys, and abnormal aspartyl and isoaspartyl residues on age-damaged proteins [see (Grillo and Colombatto, 2005) for a comprehensive overview].

One well-studied case is leucine methylation of mammalian protein phosphatase 2A (PP2A), which is involved in carbohydrate, amino acid, and lipid metabolism, and cell cycle control. PP2A is a dimeric core enzyme, composed of a structural A and catalytic C subunits, and a regulatory B subunit. The catalytic C subunit is methylated by leucine carboxylmethyltransferase-1 (LCMT-1). This is a reversible process, and the removal of the C-terminal methyl group is catalyzed by protein phosphatase methylesterase 1 (PME1) (Lee and Stock, 1993; Xie and Clarke, 1994; Ogris et al., 1999; Wandzioch et al., 2014). A crystal structure of human LCMT-1 shows that it contains a canonical SAM-dependent methyltransferase (MT) domain and a unique lid domain composed of α helices. The lid domain forms a deep active-site pocket that presumably binds to the carboxyl terminus of the PP2A tail (Stanevich et al., 2011). Interestingly, the extensive contacts between PP2A active site and LCMT-1 are essential for methylation of the PP2A tail. This mechanism suggests that efficient conversion of activated PP2A into substrate-specific holoenzyme minimizes unregulated phosphatase activity or formation of inactive holoenzymes (Stanevich et al., 2011). The active form of PP2A is critical for dephosphorylation of tau protein, which plays a crucial role in maintaining microtubule stability (Barbier et al., 2019). Deficiency of methylation on the catalytic subunit of PP2A has been associated with tau hyperphosphorylation, which leads to its aggregation into neurofibrillary tangles that correlate with the severity of phospho-tau pathology in Alzheimer’s disease (Sontag et al., 2004).

The prenylated Cys of CaaX motif undergoes methylation after the cleavage of the aaX tripeptide (Grillo and Colombatto, 2005). Methylation of prenylated Cys increases the hydrophobicity of the prenyl membrane anchor, mediating its membrane association. Evidence showed that methylation plays a very important role in localizing prenylated proteins to the membrane (Michaelson et al., 2005). Prenylcysteine methylation also enhances protein-protein interactions such as interactions between lamin B and the nuclear envelope associated proteins, as well as K-Ras association with microtubules, and Rho GTPases binding to RhoGDI (Chen et al., 2000; Maske et al., 2003; Cushman and Casey, 2009). Cys methylation is catalyzed by isoprenylcysteine carboxyl methyltransferase (ICMT), an ER membrane-associated methyltransferase. The first crystal structure of a eukaryotic ICMT was solved in 2018, using ICMT from beetle *Tribolium castaneum* (Diver et al., 2018). X-ray structure shows that ICMT contains eight transmembrane α-helices (M1-M8), and that almost the entire structure resides within the ER membrane. The active site is located mostly within the cytosolic leaflet of the membrane, and is contained between the M4 region and the C terminus (Diver et al., 2018). Two arginine residues Arg173 (on M6) and Arg246 (on M8) coordinate and position the carboxylate of the prenylcysteine substrate for catalysis, and also provide specificity for the carboxylate. Defects of methylation on prenylated cysteines affect a variety of biological processes; ICMT catalyzes methylation on terminal isoprenylcysteine of lamin A to ensure its incorporation into the nuclear envelope (Casasola et al., 2016). Inhibition of the ICMT activity results in unmethylated Ras and B-Raf, a signaling component downstream of Ras, which disrupts the transformation of cells (Bergo et al., 2004). Disruption of the methylation of Rho proteins severely impairs both random and directed cell migration (Cushman and Casey, 2011). A recent study showed that deficiency of isoprenylcysteine ICMT leads to progressive loss of photoreceptor function in mice (Christiansen et al., 2016).

### Tyrosination

Tyrosination, addition of Tyr to a protein, has been identified exclusively on tubulin, the main structural constituent of the microtubules. The microtubule lattice is formed by evolutionarily conserved α-and β-tubulin dimers, which can be modified by a broad range of functional groups. Enzymatic detyrosination and subsequent tyrosination of tubulin is a cyclic process that plays very important roles in modulating microtubule functions in mitosis, neuronal differentiation, and cardiomyocyte contraction (Aillaud et al., 2017; Nieuwenhuis and Brummelkamp, 2019) and likely affects other microtubule functions. This cyclic event happens solely on α-tubulin. Most of the α-tubulin isoforms encode a C-terminal tyrosine, except for TUBA8 that ends with phenylalanine, and TUBA4A that contains a terminus resembling detyrosinated α-tubulin (Gadadhar et al., 2017).

The detyrosination/tyrosination cycle of tubulin was discovered more than 40 years ago, following an observation that rat brain homogenate has the capacity to incorporate tyrosine into α-tubulin in a translation-independent manner (Arce et al., 1975). The enzyme responsible for this α-tubulin-specific incorporation was shortly thereafter purified and designated tubulin tyrosine ligase (TTL). While the first TTL was discovered in 1970s (Arce et al., 1975), the enzyme responsible for detyrosination was not identified until 2017, when two groups independently found that vasohibins/small vasohibin binding protein complex (VASHs/SVBP) possess the ability to remove tyrosine at the C-terminus (Arce et al., 1975; Aillaud et al., 2017; Nieuwenhuis and Brummelkamp, 2019). Biochemical studies revealed that TTL exclusively modifies unpolymerized tubulin, however, VASHs/SVBP preferentially catalyze the detyrosination step on microtubules.

TTL includes three structurally distinct domains: N-terminal (residues 1–71), central (residues 72–188) and C-terminal (residues 189–377); the enzyme’s active site is located in the C-terminal domain (Szyk et al., 2011). TTL binds to the heterodimer interface of tubulin and interacts with the major part of α-tubulin, recognizing the conformation specific to non-polymerized tubulin and absent from the microtubules (Szyk et al., 2011; Prota et al., 2013). VASHs recognize α-tubulin tail through its transglutaminase- like cysteine protease domain, which contains helical N- and C-lobes with the active site located at the interface between these two lobes. VASHs form complexes with SVBP, which stabilizes the active site of VASH (Li et al., 2019). Its specificity toward C-terminal tyrosine is determined by a serine residue (S221 in VASH1 and S210 in VASH2) and an adjacent arginine (R222 in VASH1 and R211 in VASH2), however, the preference of VASH1 and VASH2 toward microtubules is yet to be determined (Li et al., 2019). It is possible that this enzyme also acts on other Tyr-containing protein substrates.

The detyrosination/tyrosination cycle affects the binding of microtubule-associated proteins (MAPs) and the microtubule motors, kinesins, modulating their processivity (Nieuwenhuis and Brummelkamp, 2019). Through these effects, enzymes involved in detyrosination/tyrosination mediate microtubule-dependent biological processes *in vivo*. Mice lacking TTL die perinatally, with poorly developed neuronal networks, even though microtubule distribution is not grossly affected in TTL-deficient cells (Erck et al., 2005). In mice hemizygous for TTL (TTL±), reduced TTL expression, leads to a significant change in the detyrosinated/tyrosinated tubulin ratio, resulting in deficiencies in synaptic plasticity and memory; moreover, a reduced TTL level is characteristic for Alzheimer’s Disease (Peris et al., 2021). Defects in tubulin detyrosination cause structural brain abnormalities and cognitive impairments in mice, and these effects are recapitulated in human patients with familial mutations in detyrosination enzymes (Pagnamenta et al., 2019). Tubulin tyrosination-detyrosination cycle is required for stabilizing of kinesin-mediated microtubule-kinetochore attachment to promote mitotic error corrections (Ferreira et al., 2020). Inhibition of tubulin detyrosination by using parthenolide disrupts microtubule anchoring at the Z disks of the sarcomeres, leading to reduced stiffness of the cardiomyocytes (Robison et al., 2016). It has been found that Phe can incorporate into the tubulin C-terminus in place of Tyr that can also serve as a site for dopamine binding (Ditamo et al., 2016; Dentesano et al., 2018). Blocking the tubulin C-terminus to prevent these modifications interferes with the microtubule-dependent transport in neurons (Zorgniotti et al., 2021).

### C-Degrons

Protein termini can determine metabolic stability of proteins and mediate protein degradation (Varshavsky, 2019). The terminal degradation signals are called degrons. N-degrons, created by proteolytic cleavage or enzymatic modifications of the N-termini to facilitate these proteins’ turnover, have been known for a long time [e.g., (Hwang et al., 2010; Piatkov et al., 2014) for an overview]. Recently, it was discovered that stereo-chemically unique C-terminal polypeptides can serve as degradation signals, prompting the term “C-degron” (Lin et al., 2015, 2018). Such C-degrons are grouped into three different groups, including full length proteins, C-termini generated by cleavage, and prematurely terminated products (Lin et al., 2018; Varshavsky, 2019). A detailed characterization of C-degrons and related functional pathways are yet to determined (Yeh et al., 2021). It appears likely that other intracellular mechanisms could contribute to this pathway. For example, as the N-terminal arginylation can occasionally act as an N-degron; similarly, C-terminal modifications could be potential contributors to the C-degron pathway.

## Conclusion

With the development and application of mass spectrometry technology, more and more new post-translational protein modifications are being identified and emerge as previously unknown regulatory mechanisms. Modifications at both N- and C-termini of proteins make an important contribution to proteomic diversity and complexity in post-translational modifications. N- and C-terminomes play a vital role in global biological pathways including protein regulation, cytoskeleton function, cellular signaling, embryogenesis, and cell viability.

Often, modifications of the protein termini have different effects on their *in vivo* targets. For example, N-terminal arginylation affects not only the half-life of proteins through the ubiquitin mediated degradation but also the β-actin cytoskeleton which influences the cell motility (Karakozova et al., 2006; Sriram et al., 2011). C-terminal lipidation influences protein membrane localization and protein-protein interactions, and its dysfunction can lead to various diseases, such as cancer, cardiovascular diseases, neurodegeneration, and metabolic disorders (Xu et al., 2015). Collectively, investigation of the terminal post-translational modifications broadens our knowledge on protein terminome. Dysfunction and dysregulation of terminal modification enzymes lead to human diseases including cancer and neurodegenerative disorders, attracting great attention and efforts to this field.

Since terminal modifications often target the same reactive groups on different proteins, they likely exist *in vivo* in a complex interplay, in which choices between different modification can drive the metabolic fate and functions of specific proteins. Such interplay further adds to the post-translationally generated complexity of the proteome, and is virtually unexplored. We are aware of only a few examples of such interplay—e.g., actin, which is normally ∼98% N-terminally acetylated, has been discovered to also undergo arginylation at nearly the same site (Karakozova et al., 2006). Structural predictions suggest that arginylation and acetylation at the actin’s N-terminus are mutually exclusive (Rebowski et al., 2020). Indeed, direct and indirect evidence suggest that these two modifications exist in a potentially functional interplay. While abolishment of arginylation reduces cell motility and actin polymerization (Saha et al., 2010), knockout of actin acetyltransferase NAA80 facilitates these events (Drazic et al., 2018), as well as dramatically increasing the arginylated actin level (Chen and Kashina, 2019), indicating a potentially antagonistic relationship between N-arginylation and *N*-acetylation. Beyond a doubt, other terminal modifications of proteins also exhibit structural and functional interplay. Uncovering these mechanisms constitutes an exciting direction of future studies.

## Author Contributions

LC and AK wrote the manuscript and prepared the figures. Both authors contributed to the article and approved the submitted version.

## Conflict of Interest

The authors declare that the research was conducted in the absence of any commercial or financial relationships that could be construed as a potential conflict of interest.

## Publisher’s Note

All claims expressed in this article are solely those of the authors and do not necessarily represent those of their affiliated organizations, or those of the publisher, the editors and the reviewers. Any product that may be evaluated in this article, or claim that may be made by its manufacturer, is not guaranteed or endorsed by the publisher.

## Footnotes

1
https://www.nextprot.org/about/human-proteome

2
https://www.ebi.ac.uk/merops/index.shtml
